# Addressing the Child and Adolescent Mental Health Gap After the Pandemic: Why Translational, Practice-Oriented Research Matters

**DOI:** 10.3390/children12121648

**Published:** 2025-12-04

**Authors:** Ignasi Navarro-Soria, Boglarka Adorjan

**Affiliations:** Departamento de Psicología Evolutiva y Didáctica, Facultad de Educación, Universidad de Alicante, Carretera San Vicente del Raspeig s/n, San Vicente del Raspeig, 03690 Alicante, Spain; bogi.adorjan@gmail.com

The global disruption caused by COVID-19 has drawn renewed attention to an already pressing reality: child and adolescent mental health requires sustained, system-level investment and high-quality evidence to guide practice. Even before the pandemic, mental health conditions were among the leading causes of disability in young people worldwide. The Lancet Commission on Global Mental Health and Sustainable Development urged a decisive shift toward closing the science-to-service gap and scaling evidence-based interventions across systems of care [1]. The pandemic then acted as a stress test for families, schools, and services: meta-analyses and national surveillance studies showed meaningful increases in internalizing symptoms and service needs among children and adolescents, with disparities concentrated in vulnerable groups [2,3,4].

Against this backdrop, the current Special Issue brings together empirical studies and practice-oriented innovations spanning early childhood through adolescence. Collectively, these contributions foreground three cross-cutting priorities: (a) early detection and monitoring of developmental-mental health needs in real-world settings; (b) feasible, scalable supports embedded in natural ecologies (families, classrooms, community); and (c) technology-enabled solutions that complement (not replace) human relationships at the heart of mental health care.

## 1. Early Childhood: From Developmental Risk to Protective Classrooms

A consistent lesson from developmental science is that risk and protection co-accumulate over time. In early childhood, language, pragmatic communication, self-regulation, and classroom climate interact bidirectionally with socio-emotional development. Pragmatic language challenges can undermine peer relations and teacher–child interactions, while supportive, predictable classroom environments buffer risk and foster competence. Evidence accumulated over the last decade indicates that strengthening protective factors at the classroom level (e.g., warm teacher–child relationships, clear routines, emotionally attuned feedback) can yield measurable improvements in child adjustment, especially when adversity is present [5,6,7].

The studies in this collection that examine preschool communication and early years’ classroom supports align with this translational aspiration: they employ real-world samples and focus on feasible practices (e.g., coaching, structured routines, targeted small-group supports). Such work advances the field by moving beyond efficacy trials to effectiveness and implementation questions—what works, for whom, and under which classroom conditions—while keeping sight of equity considerations.

## 2. Middle Childhood and Adolescence: Executive Functions, Digital Behaviors, and Life Satisfaction

During middle childhood and adolescence, executive functions (working memory, inhibition, and cognitive flexibility) are tightly coupled with academic outcomes, emotion regulation, and risk behaviors. In that developmental window, problematic technology use can co-occur with anxiety and cyberbullying and may both reflect and exacerbate self-regulatory difficulties. Recent studies, including those represented in the Special Issue, underscore that profiles of executive functioning are not simply correlates of mental health; they are tractable intervention targets with classroom-level impact [8,9,10].

Physical education and active pedagogies appear to be promising contexts for promoting subjective well-being and life satisfaction, especially when teachers explicitly link bodily engagement with self-awareness and regulation strategies [11]. This aligns with broader evidence that school-based programs with active ingredients, emotion coaching, behavioral rehearsal, goal setting, and peer collaboration produce small-to-moderate gains in mental health and learning when delivered with fidelity in supportive climates [6].

## 3. Neurodevelopmental Conditions and Somatic Comorbidity: A Systems Lens

Neurodevelopmental disorders often present with complex constellations of somatic or connective-tissue-related symptoms, sleep disturbances, and sensory processing differences [12]. Far from being accidental, these features may shape stress physiology, pain sensitivity, and participation in learning, thereby influencing mental health trajectories. The Special Issue includes exploratory designs that invite a more integrative systems lens, one that weaves together neurodevelopmental profiles, embodied experience, and family/school accommodations. Such a lens helps translate clinical insight into practical screening, referral, and support pathways across health and education.

## 4. Digital Mental Health: Opportunity with Guardrails

Digital approaches, mobile apps, teleconsultation, and AI-enabled tools can expand reach, offer just-in-time self-help, and amplify scarce specialist capacity. Systematic meta-reviews show that digital mental health supports can be acceptable and beneficial for youth, particularly when they are human-supported, embedded in services, and underpinned by solid clinical content [13]. The Special Issue features work on mobile consultation and toolkits that exemplify this “digital-plus-human” model. Yet, the pandemic also reminded us that technology can amplify inequities and does not uniformly benefit all learners. A prudent way forward is to treat digital tools as adjuncts that enhance, not replace, relationship-based care, with attention to accessibility, privacy, and cultural fit [13,14].

## 5. Suicide Risk, Self-Harm, and the Urgency of Pathways to Care

Emergency presentations for suicidal behavior and non-suicidal self-injury rose in several settings during and after the pandemic [2,15]. Retrospective cohort approaches, like those included in this Special Issue, provide needed signals about age, sex, and contextual correlates of acute risk. However, signals must translate into pathways: (a) routine, developmentally appropriate screening in schools and primary care; (b) rapid access to stepped care; and (c) continuity across crisis, outpatient follow-up, and school re-entry. Strengthening these pathways requires intersectoral collaboration and data systems that track outcomes beyond discharge.

## 6. A Unifying Thread: Translation to Practice and Policy

What binds the contributions of this collection is not a single methodology, but a shared commitment to usable knowledge: measures that teachers can administer; classroom strategies that fit the school day; family-centered practices that respect caregiver load; and digital resources that are simple, secure, and clinically grounded. This is precisely the translational ethos advocated by the global mental health movement, connecting rigorous evidence with scalable delivery in the settings where children grow and learn [1].

At the same time, the field must address persistent evidence gaps. First, effectiveness in diverse, under-resourced contexts remains underexplored; much of the literature is still anchored in high-income settings [1,16]. Second, heterogeneity is the rule: subgroup analyses by sex/gender, neurodevelopmental profile, socioeconomic status, and cultural community are essential to avoid one-size-fits-all recommendations. Third, implementation matters: fidelity, training load, and teacher well-being are not side issues but mediators of impact.

## 7. Where Do We Go from Here?

A pragmatic agenda emerges from the studies gathered in this Special Issue and the wider literature:

1. Detect earlier, support sooner. Embed brief, valid screeners for social-emotional development, language/pragmatics, and executive functions into routine educational workflows, with clear referral pathways and feedback loops to families and teachers [5,6,9].
2. Invest in protective classrooms. Scale professional learning that helps teachers create emotionally supportive, structured, and culturally responsive environments; prioritize low-burden practices that are demonstrably linked to student mental health and engagement [6,7].
3. Couple digital with human supports. Leverage apps and teleconsultation to expand access, but ensure clinician oversight, data protection, and attention to digital equity; evaluate not only efficacy but also uptake and sustained use in everyday life [13,14].
4. Bridge health and education. Co-design stepped-care pathways that span school, primary care, and specialized services, with protocols for crisis risk, re-entry after hospitalization, and ongoing accommodations for neurodevelopmental diversity [2,15].
5. Measure what matters. Beyond symptom change, track functional outcomes: attendance, peer relationships, life satisfaction, and participation, outcomes that families, students, and teachers recognize as meaningful [11,16].

None of this is easy. But a decade of calls, from global commissions to national strategies, converges on the same point: children’s mental health is foundational to learning, development, and social cohesion [1]. The studies in this Special Issue, though diverse in scope and method, move the needle by keeping the focus where it belongs: early, feasible, relational, and equitable supports [16].
